# Expression of Genes for Si Uptake, Accumulation, and Correlation of Si with Other Elements in Ionome of Maize Kernel

**DOI:** 10.3389/fpls.2017.01063

**Published:** 2017-06-19

**Authors:** Boris Bokor, Slavomír Ondoš, Marek Vaculík, Silvia Bokorová, Marieluise Weidinger, Irene Lichtscheidl, Ján Turňa, Alexander Lux

**Affiliations:** ^1^Department of Plant Physiology, Faculty of Natural Sciences, Comenius University in BratislavaBratislava, Slovakia; ^2^Comenius University Science ParkBratislava, Slovakia; ^3^Department of Human Geography and Demography, Faculty of Natural Sciences, Comenius University in BratislavaBratislava, Slovakia; ^4^Institute of Botany, Plant Science and Biodiversity Centre of Slovak Academy of SciencesBratislava, Slovakia; ^5^Department of Molecular Biology, Faculty of Natural Sciences, Comenius University in BratislavaBratislava, Slovakia; ^6^Core Facility Cell Imaging and Ultrastructure Research, University of ViennaVienna, Austria

**Keywords:** ionome, maize kernel, PCA analysis, silicon (Si), Si correlation with elements, *ZmLsi* transporter genes

## Abstract

The mineral composition of cells, tissues, and organs is decisive for the functioning of the organisms, and is at the same time an indicator for understanding of physiological processes. We measured the composition of the ionome in the different tissues of maize kernels by element microanalysis, with special emphasis on silicon (Si). We therefore also measured the expression levels of the Si transporter genes *ZmLsi1*, *ZmLsi2* and *ZmLsi6*, responsible for Si uptake and accumulation. Two weeks after pollination *ZmLsi1* and *ZmLsi6* genes were expressed, and expression continued until the final developmental stage of the kernels, while *ZmLsi2* was not expressed. These results suggest that exclusively ZmLsi1 and ZmLsi6 are responsible for Si transport in various stages of kernel development. Expression level of *ZmLsi* genes was consistent with Si accumulation within kernel tissues. Silicon was mainly accumulated in pericarp and embryo proper and the lowest Si content was detected in soft endosperm and the scutellum. Correlation linkages between the distribution of Si and some other elements (macroelements Mg, P, S, N, P, and Ca and microelements Cl, Zn, and Fe) were found. The relation of Si with Mg was detected in all kernel tissues. The Si linkage with other elements was rather specific and found only in certain kernel tissues of maize. These relations may have effect on nutrient uptake and accumulation.

## Introduction

The composition of minerals in a cell, tissue, or the whole organism is referred to as the ionome. The ionome consists of essential and also non-essential elements, thus it represents the inorganic component of plant organism (Salt et al., 2008). Recently, increased attention has been paid to the study of the plant ionome, especially in cereals (Lombi et al., 2009; Baxter et al., 2013, 2014; Baxter, 2015; Bokor et al., 2015; Asaro et al., 2016; Shakoor et al., 2016) or other plant species (White et al., 2012; Parent et al., 2013; Pongrac et al., 2013b). Plant ionome, element profiles, distribution, and correlation among elements in various tissues may depend on environmental stimuli. For example, element tissue composition can vary greatly upon various abiotic stress conditions such as metal toxicity as it was found out in maize root and shoot (Bokor et al., 2015). In those plants, the root ionome was much more affected than the shoot ionome due to Zn exposure, and, surprisingly, also by Si application, however with lower intensity. Other metals can considerably cause alterations of the element profile in plants, as it was, for example, revealed in sensitive population of *Silene paradoxa* under Cu exposure (Pignattelli et al., 2013). Similarly, the altered distribution of elements in response to the influence of non-essential toxic element Cd in two isolates of *Salix caprea* was reported by Vaculík et al. (2012). Phylogenetic aspect is also important, because shoot ionome varies at ordinal level in angiosperms (Broadley et al., 2004). Understanding of relationships between elements is crucial to unravel the regulation of ionome. These relationships can exist regardless of the tissue, species, or environment; however, many associations will vary with a combination of these factors (Baxter, 2009). For the experiments with element tissue distribution, seeds may represent an ideal object, because the ionome of mature seeds represents a developmental end-point, showing plant life history with a combination of genetic program and interactions of environment (Baxter et al., 2014). Analysis of buckwheat seeds showed different mineral concentration and accumulation in various seed tissues, including pericarp, endosperm, cotyledons and testa with aleurone layer. Most of the measured elements were preferentially localized in cotyledons of the seed, although Ca was predominant in pericarp tissue. In cotyledons, the co-localisation of Mg and P indicated a possible binding of Mg to phytic acid (Pongrac et al., 2013b). In cereals, higher mineral accumulation is characteristic for embryo and external tissues of the grain, whereas the starchy endosperm is less enriched by minerals (Mikuš et al., 1992, 1995; Lombi et al., 2011). Different distribution of various micro- and macronutrients varied remarkably also in different tissues of rice grain, including husk, bran, endosperm, and embryo (Lombi et al., 2009). Taken together, mineral elements are not concentrated uniformly along the seed or grain, not even in a cob carrying variable amount of kernels in maize. Different accumulation of Na, S, Ca, Fe, Cu, Zn, and Sr resulted in unequal concentration of these elements in the base, middle and the tip of the corn cob in maize (Baxter et al., 2014). Concentration and accumulation of elements is the result of many physiological processes, including root uptake, remobilisation and translocation within the plant, and subsequently deposition and storage in the seed (Shakoor et al., 2016).

Silicon, as a beneficial element for plants, has been studied frequently, mainly in experiments dealing with its relation to abiotic and biotic stress. To this date, an essential function of Si has not been proven in higher plants, however, the list of beneficial effects is still increasing (for details see reviews: Liang et al., 2007, 2015; Meharg and Meharg, 2015; Líška et al., 2017). As an example, the importance of Si in increased crop yield became evident; Detmann et al. (2012) showed that higher crop yield of rice resulted from increased weight of grains after Si application. Silicon accumulation ranges from 0.1 to 10% of dry weight in plants and in many species, especially in monocots, Si uptake is an active process (Epstein, 1994; Ma et al., 2006; Currie and Perry, 2007). In maize, *ZmLsi1*, *ZmLsi2*, and *ZmLsi6* genes are responsible for direct Si transport within the plants (Mitani et al., 2009a,b). The information revealing Si concentration in various harvested portions of crops, mainly in grains, is limited. Some known data show averaged Si concentration of 2.6 g kg^-1^ in corn kernel and 23 g kg^-1^ in rice panicles, including grain and the husk (Tubana et al., 2016). In southern India, the critical limit of 12 g kg^-1^ Si was established for rice grain; below this concentration there is the likelihood of a crop significantly responding to Si fertilization (Narayanaswamy and Prakash, 2009). Ma et al. (2003) observed a large variation in grain Si concentration of barley grain ranging from 0 to 3.8 g Si kg^-1^ of dry weight; silicon was mostly localized in the hull (more than 80% of total Si). In their study, various barley varieties grown in the same field were tested, and authors suggested that variation in grain Si concentration is controlled genetically. Genotypic variation in Si concentration of grain may be associated with variation in the ability of root to take up Si, in differences in translocation and accumulation of Si, and in other factors (Ma et al., 2003).

In human physiology, silicon is an essential element, mainly important in bone formation and crucial in connective tissues. However, its bioavailability from solid foods is limited. Especially, Si bioavailability from phytolits in plant-base foods is very low (Jugdaohsingh et al., 2002 and references therein). Silicon deprivation affects collagen at different stages in bone development, collagen-forming enzymes, or collagen deposition in other tissues would have implications that Si is important for both wound healing and bone formation (Seaborn and Nielsen, 2002).

In the present study, we investigated molecular mechanisms of Si transport in maize kernel. We focused on two principal questions: which *ZmLsi* genes are expressed and where is Si accumulated in the kernel. Another aspect of this work concerns the possible correlation of micro- and macroelement with Si in various kernel tissues, including pericarp, soft endosperm, scutellum, and embryo proper. The possible role of a specific *ZmLsi* expression and possible physiological explanation for the existence of multilateral correlation patterns among Si and other elements in maize kernel tissues are discussed.

## Materials and Methods

### Plant Material and Cultivation

Maize (*Zea mays*, hybrid Novania) kernels were obtained from plants cultivated in soil under natural conditions. The plants were grown in sandy-loam soil in the yard of the Department of Plant Physiology, Comenius University in Bratislava and watered regularly from April till October 2015. No insecticides or additional foliar spraying neither addition of nutrients in the form of fertilizers were applied. The concentration of bioavailable Si from the soil (113 ± 15 mg kg^-1^) was analyzed according to Rodrigues et al. (2003) with modification. After extraction by 0.5 M acetic acid, Si was measured by ICP-MS in place of colorimetric determination using blue silicomolybdous acid procedure as used in the original procedure. The concentration of Si in other plant organs was determined by AAS (Supplementary Table S1) as described previously by Bokor et al. (2014).

Male and female flowers were protected in a bag to prevent uncontrolled pollination. During flowering, pollination was performed manually. During maize kernel development, we selected three developmental stages of kernels for analyses: 2 weeks after pollination (the 1st developmental stage), 1 month after pollination (the 2nd developmental stage), and mature dry kernel (the 3rd developmental stage).

### Real-Time PCR Analysis

RNA of the kernel from the 1st developmental stage was extracted from pericarp and the rest of the kernel, because it was possible to extirpate the embryo at this stage. At the 2nd developmental stage, RNA was extracted from pericarp, embryo, and the whole kernel. At the 3rd developmental stage, RNA was extracted from the pericarp and embryo. The Real-time PCR analysis was performed according to our previous work (Bokor et al., 2015).

Briefly, total RNA was extracted and DNase I treated using Spectrum Plant Total RNA kit (Sigma–Aldrich). In addition, RNA extraction of the kernel in the 3rd developmental stage was done after 24 h imbibition in sterile conditions. Synthesis of cDNA was performed by ImProm-II Reverse Transcription System (Promega) using Oligo(dT)15 primers and after that, cDNA was purified by DNA Clean & Concentrator^TM^-5 (ZymoReseach). *ZmLsi1*, *ZmLsi2*, *ZmLsi6*, and *beta actin* genes were amplified by Maxima SYBR Green/ROX qPCR Master Mix (Thermo Fisher Scientific) using Light Cycler II 480 (Roche). The relative changes in gene expression were estimated according to Pfaffl method including amplification efficiency of selected genes (Pfaffl, 2001). After the collection, the samples were stored in -80°C before analysis. All samples for PCR experiments were analyzed in three biological and technical replicates.

### Scanning Electron Microscopy (SEM) Coupled with X-ray Microanalysis

**FIGURE 1:**
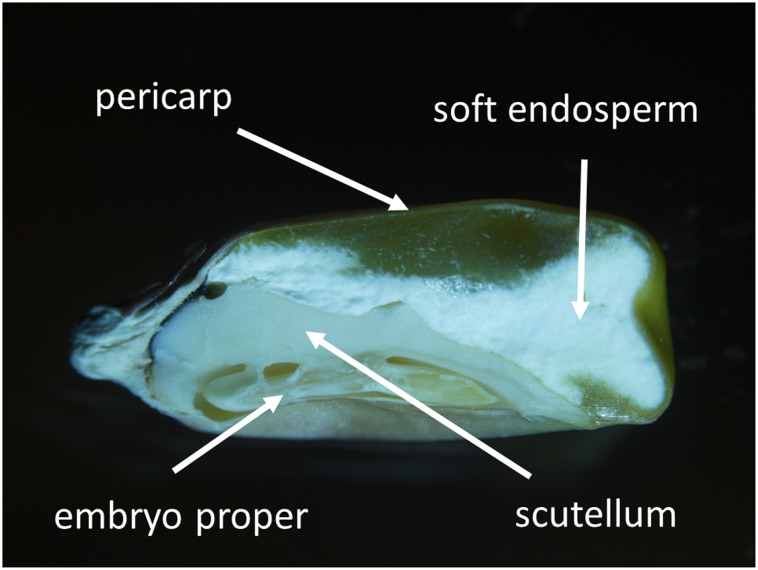
Longitudinal section of mature maize kernel (the 3rd developmental stage). Kernel tissues including pericarp, soft endosperm, scutellum, and embryo proper are indicated.

Longitudinally sectioned and air-dried mature maize kernels (the 3rd developmental stage) were coated with carbon and fixed on aluminum stubs covered with a carbon sticker. Surface conductivity was increased by carbon coating resulting in a uniform thickness of the carbon layer of approximately 60 nm. Kernel tissues (**Figure 1**) and the distribution of 11 elements (N, Zn, Ca, K, P, S, Si, Mg, Cl, Cu, Fe) were observed and analyzed with the Jeol JSM-IT300 scanning electron microscope (SEM) equipped with an energy dispersive X-ray (EDX) analyser. Initial mapping showed relatively low differences in element distribution within kernel tissues (**Supplementary Figure S1**), therefore for relative quantification of element distribution all samples were observed at 1000x magnification and 20 kV accelerating voltage, working distance was 11.0 mm. Data were collected for 50 live seconds. As uneven surfaces may heavily distort EDX measurements (Goldstein, 2003), only flat parts of the samples were selected for analysis. For EDX measurements, three biological and four technical replicates were performed.

In total, four different mature maize kernels were investigated by measuring four different spots on each of four maize kernel areas (pericarp, soft endosperm, embryo proper, and scutellum). This indicates that data for statistical processing were obtained from 16 individual measurements of each maize kernel zone. Raw data were processed with the TEAM Enhanced, Version 4.3 (EDAX-Ametek, United States) software and all the values are expressed as weight % of total analyzed elements.

### Statistical Analysis

Statgraphics Centurion (version 15.2.05) software was used for statistical evaluation. The statistical significance of the means was considered at 0.05 probability level. Analysis of variance (ANOVA) and least significant difference (LSD) were performed on all experimental data sets. Additionally, standard principal component analysis (PCA) and multivariate regression analysis were applied to the study of ionome using Microsoft Excel 2013 and R version 3.0.2 software.

The data from EDX measurements were used for the study of ionome. Ionome analysis was first performed by PCA. The original data matrix consisted of 11 element variables (Ca, Cl, Cu, Fe, K, Mg, N, P, S, Si, and Zn) measured in 63 observations differentiated among four different tissue categories (embryo proper 16, soft endosperm 16, scutellum 15, pericarp 16). Principal components analysis was performed by singular value decomposition of the centered and scaled data matrix using the function “prcomp” in R version 3.0.2. Variable loadings revealed the correlation structure between original variables and extracted components. Multivariate regression analysis was performed by ordinary least squares estimation procedure “lm” in R version 3.0.2. Structural category embryo proper and Si element were assigned as references, to which all remaining categories and elements, as well as their two-way interaction terms were compared. The resulting model was statistically significant (*F*-statistic 420.9 on 43 and 649 degrees of freedom, *p*-value 0.00) with adjusted *R*-squared at the level 0.96.

Additional insights to standard statistical procedures was found in the analysis of network representation of ionome with focus on Si. Pair-wise conditional probabilities were constructed from mutual above-average element contents detected in corresponding tissue category. This approach is inspired by the network of relatedness between products of national economies, so called “product space” (Hidalgo et al., 2007), identifying conceptual analogy between international trade linking geographical territories with biological transport of elements between different tissues. Each observation was assigned above- or below-average element content, based on relative share from total sums of all element variables. Conditional probabilities corresponded to the empirical frequencies of mutual source and target elements above-average divided by the frequency of source element above-average. An oriented weighted network is created in each tissue. This method enables insights in a complexity of relationships between elements, a formation of tissue-specific clusters of elements, a hierarchy arising between the central and the peripheral roles in maize kernel ionome. For purpose of visualization we generalized full network in the fragment above threshold probability of 0.50 to focus our attention on Si and its relation to other elements in different maize kernel tissues.

**Table 1:** Macro- and microelement content expressed as weight % of total analyzed elements in mature maize kernel (the 3rd developmental stage).

	Mg	Si	P	S	Cl	K	Ca	Fe	Cu	Zn	N
Embryo proper	0,312 ± 0,022 a	0,078 ± 0,011 a	0,489 ± 0,087 a	0,148 ± 0,019 b	0,117 ± 0,013 a	0,382 ± 0,064 b	0,071 ± 0,009 b	0,081 ± 0,009 b	0,056 ± 0,009 c	0,044 ± 0,009 b	7,54 ± 0,3 a
Scutellum	0,059 ± 0,019 c	0,033 ± 0,012 b	0,092 ± 0,023 b	0,083 ± 0,015 c	0,131 ± 0,022 a	0,822 ± 0,098 a	0,191 ± 0,057 a	0,157 ± 0,032 a	0,152 ± 0,025 a	0,135 ± 0,024 a	4,20 ± 0,26 c
Soft endosperm	0,006 ± 0,003 d	0,030 ± 0,006 b	0,059 ± 0,009 b	0,202 ± 0,017 a	0,101 ± 0,016 a	0,150 ± 0,014 c	0,079 ± 0,01 b	0,094 ± 0,012 b	0,121 ± 0,017 ab	0,109 ± 0,020 a	5,65 ± 0,16 b
Pericarp	0,141 ± 0,019 b	0,103 ± 0,012 a	0,149 ± 0,013 b	0,230 ± 0,02 a	0,120 ± 0,016 a	0,225 ± 0,061 bc	0,066 ± 0,005 b	0,069 ± 0,006 b	0,077 ± 0,01 bc	0,061 ± 0,006 b	5,78 ± 0,29 b

*Values are means ± standard error. Different letters in column indicate significant differences between the treatments at 0.05 level.*

## Results

### Gene Expression Level

**FIGURE 2:**
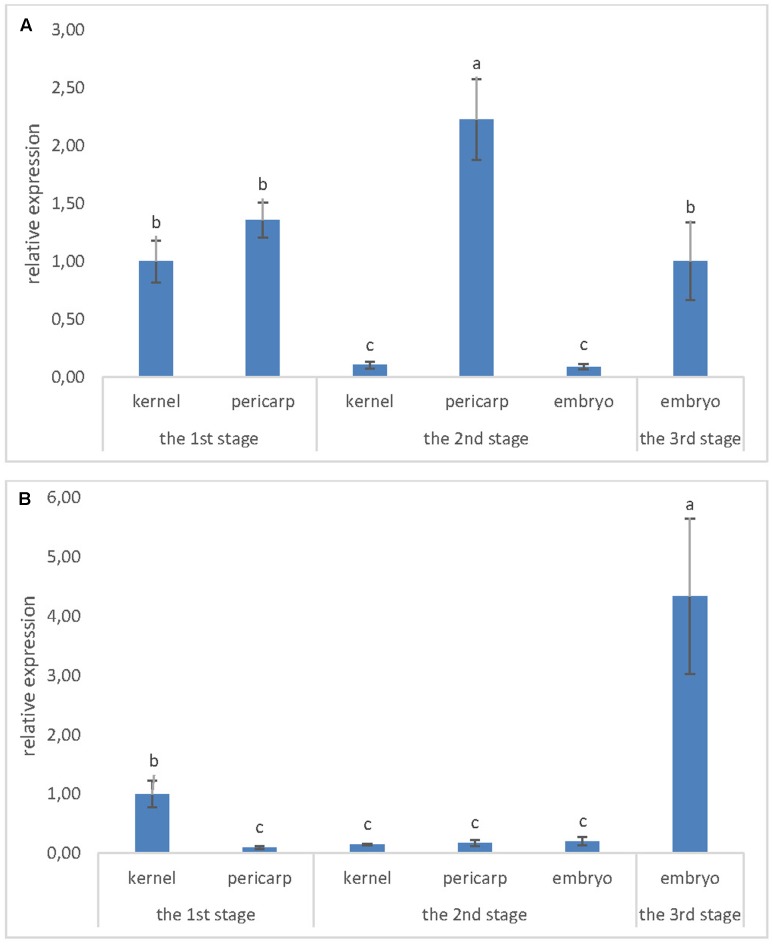
Expression level of **(A)** *ZmLsi1* and **(B)** *ZmLsi6* genes determined by real-time PCR in whole kernel or kernel tissues pericarp and embryo. The x-axis represents various developmental stages of maize kernel and related kernel tissues. Values are means ± SE. Different letters indicate significant differences at 0.05 level.

Expression of *ZmLsi* genes was detected in maize kernel at the 1st developmental stage 2 weeks after pollination (**Figure 2**). In this developmental stage, *ZmLsi1* and *ZmLsi6* genes were expressed; but the *ZmLsi2* gene was not. The same pattern of gene expression was observed also in older kernels. In the 2nd stage, only *ZmLsi1* and *ZmLsi6* were expressed in pericarp, embryo and also in the whole kernel containing endosperm tissue (**Figure 2**). In the 3rd developmental stage, pericarp tissue was senescent, thus total RNA was not possible to extract. Expression of *ZmLsi1* and *ZmLsi6* genes was found in the embryo; however, also at this stage *ZmLsi2* was not expressed. Analysis of *ZmLsi* genes corresponds with Si accumulation in adult kernel (the 3rd developmental stage). EDX analysis showed the highest Si accumulation in embryo proper and pericarp. However, very low Si content was also detected in soft endosperm and scutellum of the embryo (**Table 1**).

### Ionome Analysis

Energy dispersive X-ray measurements showed significantly higher concentration of the macroelements Mg, P, and N in the embryo proper in comparison to all other kernel tissues (**Table 1**); while the microelements Zn and Cu showed the lowest content found in the embryo proper. In the scutellum, microelements Zn, Cu, Fe and macroelements Ca and K were significantly higher when compared to the other kernel tissues. Additionally, Ca did not significantly differ between embryo proper, soft endosperm, and pericarp. Soft endosperm tissue was characteristic for very low Mg, P, K, and N content, although some elements (S, Zn, and Cu) were higher, when compared mainly with the embryo proper. In the pericarp, the concentration of microelements Fe, Cu, and Zn did not differ from values found in embryo proper. Pericarp and soft endosperm contained the highest amount of kernel sulfur. Nitrogen accumulation reached similar values between scutellum and pericarp. Preferentially, N was accumulated in the embryo, while the lowest content was found in soft endosperm.

**FIGURE 3:**
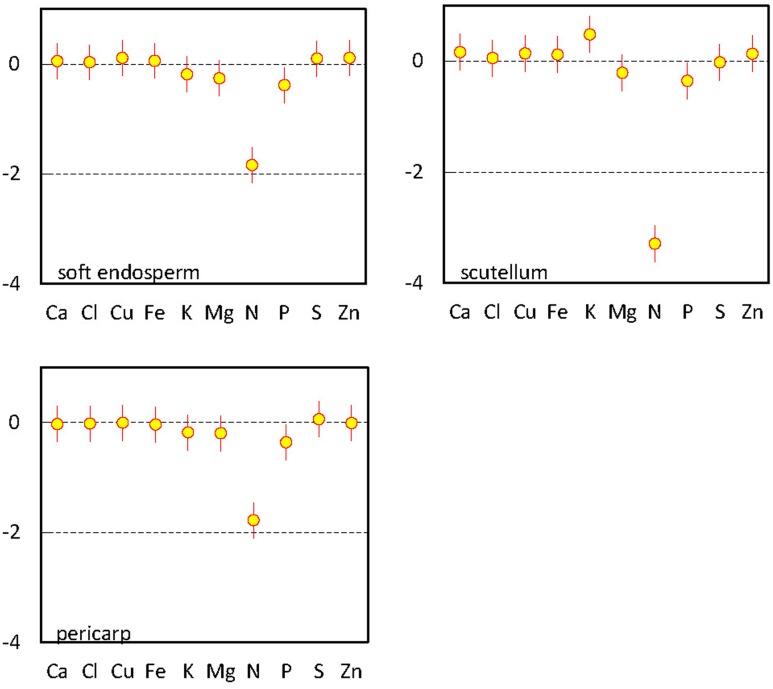
Multivariate regression analysis of elements in mature maize kernel (the 3rd developmental stage). Parameter estimates for interaction terms between tissue variables for soft endosperm, scutellum, and pericarp and remaining elements compared with reference variable (Si) in embryo proper, including 95% confidence interval bars.

Multivariate regression analysis revealed characteristic ionome changes in soft endosperm, scutellum, and pericarp tissues using comparison with Si and embryo proper as a reference category (**Figure 3**). Only three elements have significantly different content than Si detected in embryo proper, namely macronutrients K, P, and N. Considering differences between embryo proper and other tissues, content of N and P was significantly below the content of Si in embryo proper in all three remaining categories. Potassium is significantly elevated only in scutellum tissue in comparison to embryo proper (**Figure 3**). Remaining elements in the scutellum, soft endosperm and pericarp showed statistically indistinguishable contents in comparison to reference element in embryo proper in the presence of N; its content varies at a substantially higher order of magnitude and shadows variation among other elements.

**FIGURE 4:**
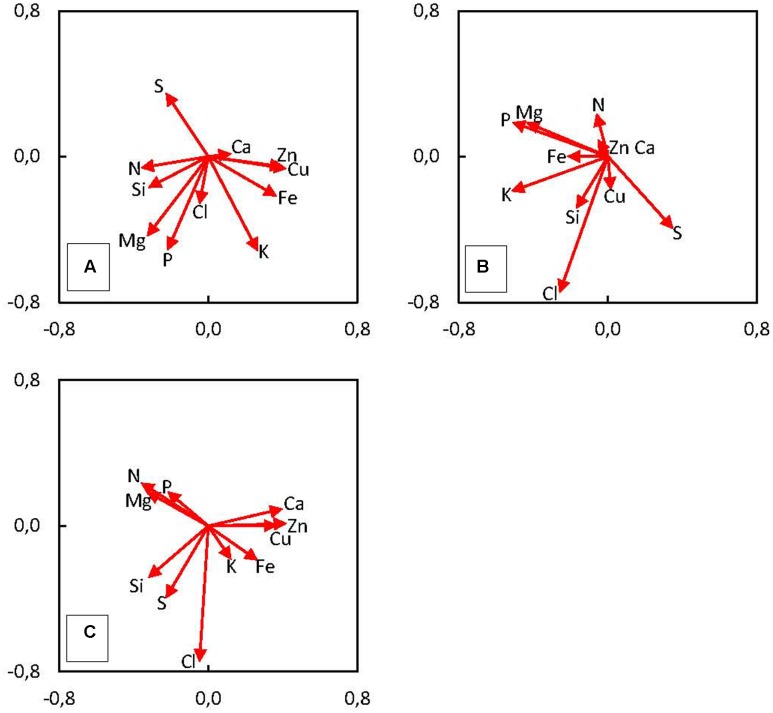
Principal components analysis (PCA) shows the correlation of measured variables (content of elements in mature kernel tissues – the 3rd developmental stage) to principal components **(A)** PC1 and PC2, **(B)** PC2 and PC3, **(C)** PC1 and PC3. The length of arrows (eigenvectors) represents the strength of correlation with the PCs. The angle between eigenvectors represents the correlation among elements.

In order to introduce additional aspect to ionome analysis, we used standard PCA, capable to determine relationships between specific tissues (pericarp, soft endosperm, scutellum, and embryo proper) and varying element composition in maize kernel (**Figure 4**). The values of mineral content were set as source variables and used for PCA. This method revealed three significant principal components (PC); PC1, PC2, and PC3 which explain 69.6% of total variance in maize kernel ionome. The PC1 (*SD*: 2.13) accounts for 41.2%, PC2 (*SD*: 1.33) accounts for 16.1% and PC3 (*SD*: 1.16) accounts for 12.3% of total variance. Projection of eigenvectors in plane defined by PCs reveals complex correlation structure in ionome. Acute angle between eigenvectors of certain elements represents a positive correlation, obtuse angle shows negative correlation and 90° angle refers to mutually uncorrelated elements. Thus, clustering of certain elements displays elemental relationships effectively.

**FIGURE 5:**
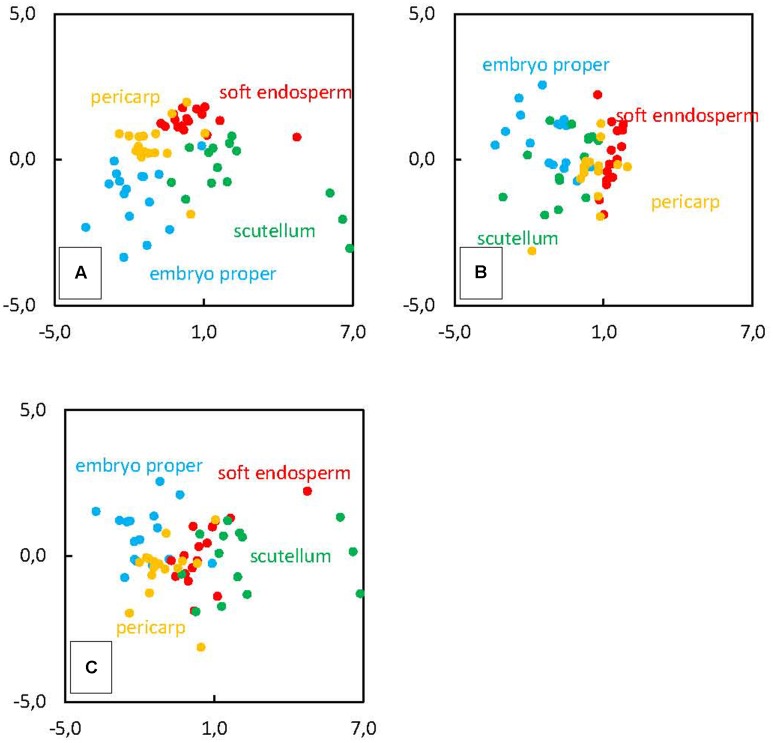
Principal components analysis of element distribution in mature kernel tissues (the 3rd developmental stage) pericarp, soft endosperm, scutellum and embryo proper. Original observations are projected into PCs score of **(A)** PC1 and PC2, **(B)** PC2 and PC3, **(C)** PC1 and PC3.

We have found positive correlation of Cu, Zn, Fe, and K and negative correlation of N, Si, Mg, S, and P with the PC1 (**Figure 4A**). The PC2 is positively correlated only with S, negatively correlated with K, P, Mg, Cl, and Fe (**Figure 4B**). Finally, PC3 is positively correlated with N, Mg, and P and negatively correlated with Cl, S, and Si (**Figure 4C**). In the case of Si clustering with other elements, correlation was found between Si and N, Mg, and P (**Figure 4A**), the other correlation represents Si and Cl together with Cu (**Figure 4B**) and the last cluster consists of Si and S and Cl (**Figure 4C**). Projection of observations in plane defined by PCs suggests the existence of underlying regularity, which is clearly tissue related, as location of four categories mostly remains well-defined in these plots, especially in **Figure 5A**, which is a plane defined by two most significant PCs. Hence, this demonstrates different accumulation of elements in various kernel tissues. Higher scores of PC1 correspond with observations categorized in soft endosperm and scutellum, lower scores of PC1 correspond with observations categorized in embryo proper and pericarp (**Figure 5A**). Concerning PC2 as a dimension orthogonal with the former one, higher scores of PC2 correspond with observations categorized in soft endosperm and pericarp, lower scores of PC2 correspond with observations categorized in embryo proper and scutellum (**Figure 5B**). Observations are clustered in four quadrants of the scatterplot PC1 vs. PC2. Third dimension is not offering any similar clustering of observations, therefore the interpretation seems to be less tissue related and more hidden in complex multilateral relationships linking together groups of elements by specific functional routes (**Figure 5C**).

**FIGURE 6:**
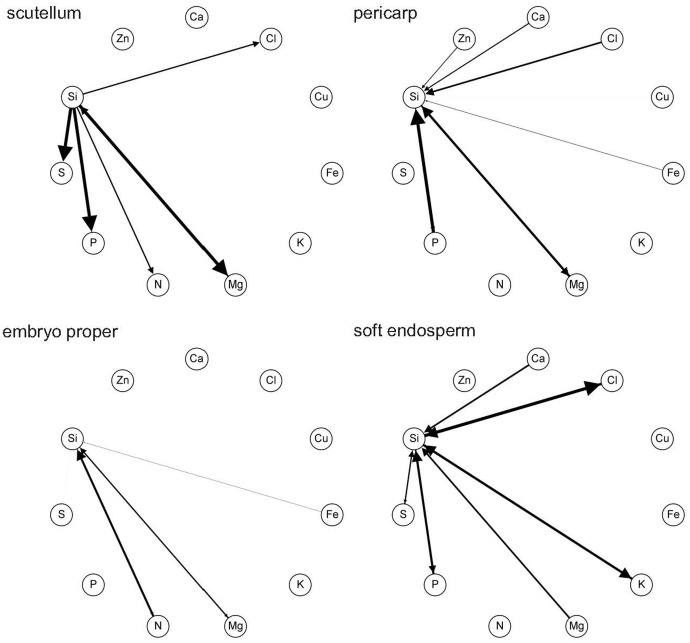
Network representation using pair-wise probabilities of mutual above-average element content in tissues: pericarp, soft endosperm, scutellum, and embryo proper of mature maize kernel (the 3rd developmental stage). This figure shows all probabilities above threshold 0.50.

Generalized network representation of ionome in kernel tissues is a novel analytical tool, proposed in order to reveal the structure of multilateral relationships within ionome equilibrium, which can be visualized similar to correlation wheels between pairs of elements. However, unlike standard correlation, pair-wise probability of mutual above average content is not necessarily reciprocal, nor even bidirectional (notice different size of arrow on a link between two elements). Thus, this method reveals varying probability patterns between different tissues corresponding with different functional relationships among elements. For example, in soft endosperm, the most evident reciprocal probability is detected between Si and Cl. It means, that if Si has above average content, there is relative high certainty that also Cl has above average content (**Figure 6**), and vice versa. However, the other connected elements, Si and S are detected with above-average content with less certainty in soft endosperm, because the correlation is weak (**Figure 6**). In scutellum, main correlations consist of Si and Mg, P, and S (**Figure 6**). In general, tissue of embryo proper contains weaker network of Si-elements relationships in comparison to scutellum, soft endosperm, and pericarp. Here, the most evident relation represents Si and N, the rest of Si relations are much weaker. In pericarp, Si is strongly correlated with P and Mg (**Figure 6**). The other correlation in pericarp represents weak Si correlation with Cl, Ca, Zn, and Cu.

The relation between Si and Mg is found in every kernel tissue, however this correlation is unidirectional only in soft endosperm (**Figure 6**). Interestingly, Si and P interaction is found only in soft endosperm, scutellum and pericarp tissue and is missing in embryo proper, where it is below threshold. In general, the most abundant relation exists between Si and Mg, P, and Cl. These findings are also supported by PCA, where these relations are clustered with Si, although Si and Cu correlation found in PCA is showed by network representation only in pericarp (weak relation, graphically not visible). Considering all kernel tissues, Si was in relation with macroelements consisting of N, P, S, Ca, and Mg and microelements including Fe, Zn, and Cl.

## Discussion

The novelty of this work represents the evidence of *ZmLsi* genes expression in developing maize kernel. It is surprising, that *ZmLsi1* and *ZmLsi6* expression occurred in maize kernel (especially embryo and pericarp), while the *ZmLsi2* gene was not expressed during kernel development at all. It is known that Lsi1 and Lsi6 transporters (NIP subfamily of aquaporins) are not energetically dependent in comparison to different property of Lsi2 transporter that needs energy to maintain its transport function (Ma et al., 2007; Zangi and Filella, 2012). The relation of Lsi1 and Lsi6 transporters to aquaporins may explain the importance of water transport during the life cycle of kernel: during early stages of its development and growth, water is very important to the point, when kernel starts dehydration process and dormancy. After imbibition of mature kernel, again, water uptake is essential for germination. Thus, the expression of aquaporins may be essential in these processes and this may be the reason for increased *ZmLsi1* and *ZmLsi6* expression. Since these transporters are capable of Si transport, Si is accumulated in kernel tissues, mainly in embryo and pericarp. As embryo is the most important part of the maize kernel and will later develop into the new organism, the expression of both *Lsi* genes in this specific region also suggests the importance of Si for optimal growth and development of maize already from early stage of plant development, i.e., embryogenesis. Additionally, increased Si accumulation in pericarp may also indicate protective function against various mechanical and biotic stresses.

Statistical analyses including PCA and network representation of elements uncovered specific correlations and/or relations of Si with other micro- and macroelements in maize kernel. Relationship of Si with Mg was found in all kernel tissues. This interaction is based on promoted uptake of Mg and also Ca by Si presence (Islam and Saha, 1969). Overally, Si related with N, P, S, Ca, Mg, K and also Cl, Fe, and Zn in the kernel. Very high positive correlations were found between Si and Mg, K, and S in reproductive organs of crops (Monti et al., 2008). Synergic effect of Si between N, P, and Ca is responsible for increased concentration of these macroelements in plants, after Si treatment (Mali and Aery, 2008a). In wheat, lower addition of Si increased concentration of macroelements K and Ca related with improved growth under stress conditions, mainly drought stress (Mali and Aery, 2008b). However, in rice plants, Si caused decrease in uptake of N, K, and Fe (Islam and Saha, 1969). Anyway, these opposing data are consistent with our results, because, e.g., in scutellum, if Si is increased, there is high probability that also N is increased (thus, Si promotes N uptake, because of arrow direction from Si to N). However, in embryo proper, if Si is increased it means that N is increased with lower probability than in reciprocal relation (if N is increased there is a high probability that Si is increased; thicker arrow direction from N to Si). The cations Ca, Mg, and K share similar chemical properties that is also responsible for interaction between them (Fageria, 2001), thus this may be the reason why Si is related with these elements. In case of microelements, Si application increases Zn and Fe content in seeds of rice (Mehrabanjoubani et al., 2015), although did not influence the concentration and tissue distribution of Zn in cucumber (Bityutskii et al., 2014). However, in maize roots and shoots, Si caused decrease in Zn concentration indicating antagonistic effect of Si (Bokor et al., 2014). Negative correlation of Cl and Si was found in crops by Monti et al. (2008). Thus, our findings support the idea, that not only ionome, but also relation of Si with other nutrients is specific, and, that Si has impact on nutrient uptake and accumulation within kernel tissues. The predominant computed synergic effect (based on pair-wise probability of mutual above average content) of Si with other nutrients may indicate its positive role in kernel tissues to promote nutrient accumulation important in seed metabolism and physiology.

In maize kernel tissues, measurements of micro- and macroelement accumulation showed interesting results. Correlation between elements in grain or seed may point to common molecular mechanisms of uptake and metabolism of these elements, or it may represent common adaptation to environment (Vreugdenhil et al., 2004). The elements Mg and P, clustered in PCA were mostly accumulated in the embryo proper. Co-localisation of Mg and P in cotyledons of the embryo was supported also by a high correlation coefficient of these elements in the pseudocereal buckwheat (Pongrac et al., 2013b). Since phytic acid is the main storage molecule of P and strongly binds Mg (and also other elements), this clearly suggests, that this correlation represents an association of Mg – P within phytic acid (Mikuš et al., 1992, 1995; Veiga et al., 2006; Persson et al., 2009; Kumar et al., 2010; Pongrac et al., 2013b). A cluster of Zn, Cu, and Fe is also detected by PCA and network of elements (however, not in soft endosperm), which proposes a shared mechanism of accumulation. Such clustering was recently found in sorghum grain and also in wheat, where the similarity between the distribution of Cu and Zn was shown (Lombi et al., 2011; Shakoor et al., 2016).

Sulfur is an element specifically included in storage proteins that may be located in cotyledons as well as in testa and aleurone of buckwheat (Pongrac et al., 2013b). Thus, N and S assimilation is coordinated (Zhao et al., 1997). We found also correlation in PCA, however, not so strong. In wheat, S can be strongly partitioned in the subaleurone tissue (Pongrac et al., 2013a). In rice grains, S occurred together with Zn and was accumulated not only in the aleurone/pericarp tissues but also in the endosperm (Lombi et al., 2009). This is similar to our results, where both Zn and S content was increased in the soft endosperm tissue in comparison to the embryo proper. Thus, complexes of Zn with –SH groups of phytochelatines and metallotioneins are indicated, although this role in cereal grains is still not fully understood (Lombi et al., 2009). In our study, we did not observe cluster Zn – S in PCA analysis, thus more investigation is needed.

Nitrogen is localized preferentially in the embryo proper of the kernel. Increased content of N may be linked to increased protein synthesis. Scutellum and pericarp contain less N than embryo proper, and soft endosperm shows the lowest content of N. Nitrogen supplementation is key component in protein synthesis and its supplementation increases total protein content in grain (Triboi et al., 2000). Positive correlation of N and Si was also supported by PCA. Silicon improves N use efficiency and enhances vegetative and generative biomass production in wheat plants grown under various Si treatments. However, grain yield nitrogen use efficiency was positively affected only by higher doses of Si (Neu et al., 2017). The highest concentration of proteins, which are metabolically active is found in embryo and less in aleurone layer. In endosperm, protein concentration is generally low and most of them represent storage proteins (Lasztity, 1995).

Mineral elements Zn, Cu, Fe, Ca, and K were higher in the scutellum in comparison to other tissues, however, Zn and Cu did not differ significantly from soft endosperm. In buckwheat, Zn, Cu, Fe, and K were preferentially localized in cotyledons of the embryo and Ca was abundant in the pericarp (Pongrac et al., 2013b). In rice grain, Zn and Cu content increased in the embryo and Fe was mainly localized in external parts of the grain (Lombi et al., 2011). The maize pericarp did not show increased micro- and macroelement accumulation in comparison to the rest of kernel tissues, except of S and Si.

The PCA analyses showed, that the elemental composition is specific for the different tissues of the kernel; it further demonstrated a regularity of mineral content in each kernel tissue. Therefore, it could be concluded that each tissue is represented by its own ionome. In a case of Si, we found several correlations of Si and other elements (the macroelements Mg, N, P, Ca, K, and S and the microelements Cl, Fe and Zn in network representation and PCA) that are also tissue specific, except of Si and Mg relation present in all tissues of maize kernel. Considering the fact that many beneficial roles of Si so far are not understood, research about its correlation with other elements in the various tissues becomes especially important. However, additional investigation is necessary to find out the background and role of this correlation in the maize kernel ionome.

## Conclusion

We found that high accumulation of Si was detected in the embryo proper and the pericarp what is supported by expression of *ZmLsi* genes in these tissues. During the developmental stages of kernel, only *ZmLsi1* and *ZmLsi6* genes were expressed, but*ZmLsi2* gene not at all. In general, the analysis of the kernel ionome showed that element correlations may be tissue specific and that each investigated tissue (pericarp, soft endosperm, embryo proper, and scutellum) is characteristic by its own ionome with more likely different level of element distribution regulation. The analyses of Si linkage to other elements showed mainly synergic effect (based on probability of above-average content of Si and other element in network representation model) on nutrient accumulation in the kernel tissues. Silicon, as important component of the maize ionome, mainly related with Mg non-specifically in all tissues. The other macroelements P, S, N, K, and Ca related with Si in at least two kernel tissues showing higher degree of tissue specificity. Similarly, Si linkage to microelements Cl, Fe, and Zn was rather tissue specific. These results show that nutrient accumulation in maize kernel may be affected by silicon.

## Author Contributions

BB designed the research, performed real-time PCR and wrote the article; SO performed statistical analysis including PCA, multivariate regression and network representation; MV performed EDX analyses and contributed to the design of research and figure preparation; SB considerably contributed to the real-time PCR experiments and analysis; MW and IL enabled EDX analyses; JT and AL supervised the project; and all authors discussed the results and commented on the article.

## Conflict of Interest Statement

The authors declare that the research was conducted in the absence of any commercial or financial relationships that could be construed as a potential conflict of interest.

## Supplementary Material

FIGURE S1 Informative map of element distribution within kernel tissues investigated in this study using SEM coupled with EDX-microelement analysis. Red frame denote the zone of maize kernel that was analyzed and distribution of each investigated element (11 in total) is marked by different color.Bar = 500 μm.
