# Supplementary figures and images for: Expression of Genes for Si Uptake, Accumulation, and Correlation of Si with Other Elements in Ionome of Maize Kernel
Source: Front Plant Sci. 2017 Jun 19;8:1063. doi: 10.3389/fpls.2017.01063 (PMC5474966; doi:10.3389/fpls.2017.01063)

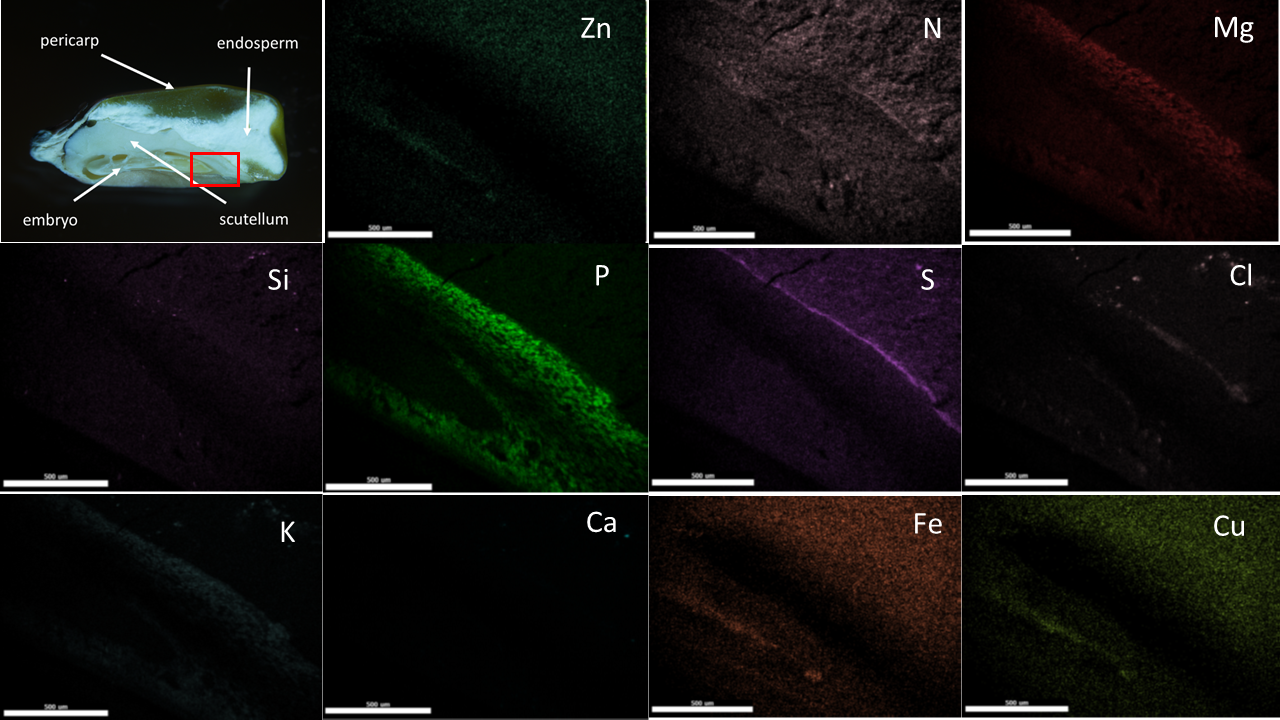

Supplement: FIGURE S1 — Informative map of element distribution within kernel tissues investigated in this study using SEM coupled with EDX-microelement analysis. Red frame denote the zone of maize kernel that was analyzed and distribution of each investigated element (11 in total) is marked by different color.Bar = 500 μm. [file Image_1.TIF]
