# Supplementary material for: Expression of Genes for Si Uptake, Accumulation, and Correlation of Si with Other Elements in Ionome of Maize Kernel
Source: Front Plant Sci. 2017 Jun 19;8:1063. doi: 10.3389/fpls.2017.01063 (PMC5474966; doi:10.3389/fpls.2017.01063)
Supplement: Supplementary file 2 [file Table_1.docx]

|  | leaf | kernel |
| --- | --- | --- |
| Si concentration (mg kg^-1^) | 22,500 ± 832 | 245 ± 35 |

Supplementary Tab. 1. The Si concentration of leaf and kernel in maize (*Zea mays*, hybrid Novania) detected by ICP-MS. The data are shown as means ± SE.

Determination of Si in plant samples:

The concentration of Si in the dry biomass of leaves and kernels was determined by atomic absorption spectroscopy (AAS). Plant samples were dried at room temperature and grinded to small pieces (<1 mm). Digestions of plant samples were carried out in the stainless steel coated PTFE pressure vessels ZA-1 (Czech Republic). Plant sample of 0.1-0.5 g was weighted to the vessel and 5 ml of concentrated HNO3, 0.25 ml of concentrated HF and 2 ml of 30 % H_2_O_2_ were added for Si detection. Vessels were closed and heated in the electric oven at 160 °C for 6 h. After digestion, 2 ml of saturated solution of H_3_BO_3_ was added and then the solution was diluted to 25 ml with redistilled water and stored in a 100-ml polyethylene (PE) bottle. Silicon concentrations were determined by flame atomic absorption spectrometry (AAS Perkin Elmer Model 5000, wavelength 251.6 nm, flame:acetylene-N_2_O).
